# W/O/W Emulsions as a Strategy to Preserve NADES-Derived Annatto Carotenoids and Enhance the Functional Properties of Potato Starch-Based Active Packaging Films

**DOI:** 10.1021/acsomega.6c04826

**Published:** 2026-07-31

**Authors:** Sara K. Balabram, Fernanda P. Silva, Bianca C. Maniglia, Larissa Tessaro

**Affiliations:** University of São Paulo, São Carlos Institute of Chemistry, Av Trabalhador São Carlense, 400, 13566-590 São Carlos, SP, Brazil

## Abstract

This study investigated the use of a water-in-oil-in-water (W/O/W) double emulsion to encapsulate a natural deep eutectic solvent (NADES)-based annatto (*Bixa orellana* L.) seed extract and incorporate it into potato starch-based films. The aim was to improve the stability and functionality of the extract compared with its direct addition to the starch matrix. The physicochemical properties of the W/O and W/O/W emulsions were first characterized, followed by the evaluation of the resulting films. The droplet size of the W/O/W emulsion (1.21 ± 0.03 μm) was larger than that of the W/O emulsion (0.42 ± 0.03 μm). The W/O/W emulsion exhibited a high encapsulation efficiency (95.5 ± 0.5%) and maintained a substantial fraction of the antioxidant activity of the encapsulated extract. When incorporated into potato starch films, the W/O/W emulsion reduced the moisture content by 46.7% and water vapor permeability by 52.2%, from 1.84 ± 0.41 to 0.88 ± 0.02 g mm m^–2^ h^–1^ kPa^–1^. The film containing the double emulsion (DE-5%) exhibited a tensile strength of 21.84 ± 0.97 MPa and showed antioxidant activity comparable to the free extract, with a measured value of 4.88 ± 0.28 mg TE g^–1^ of annatto seed. In addition, the ultraviolet–visible (UV–vis) light barrier properties were improved. Microstructural, X-ray diffraction (XRD), and thermal analyses indicated that the W/O/W emulsion modified film morphology and reduced matrix crystallinity without impairing film formation. Overall, the results indicate that W/O/W emulsions are a promising strategy to enhance the protection of NADES-derived bioactive compounds and improve the structural and functional properties of potato starch-based films for active biodegradable packaging applications.

## Graphical abstract

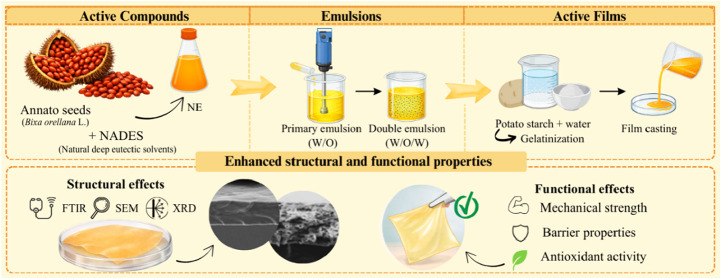

## 1 Introduction

Starch-based films have been widely investigated as sustainable materials for active food packaging because of their biodegradability, low cost, availability, and good film-forming ability.^1,2^ Among starch sources, potato starch is particularly attractive due to its intrinsic phosphate groups and molecular structure, which can favor the formation of transparent and relatively resistant films.^2,3^ However, native starch films are still limited by their high sensitivity to moisture and weak barrier performance, which restrict their practical application and justify the search for strategies capable of improving their functionality.^3,4^

One of the most explored approaches is the incorporation of plant-derived bioactive compounds, especially phenolics and carotenoids, into biopolymer matrices to confer antioxidant and light-protective functions to the material.^5–7^ Annatto (*Bixa orellana* L.) seeds are an important natural source of carotenoids, especially bixin and norbixin, and have attracted interest because of their antioxidant activity and potential use in active materials.^8^ However, direct incorporation of these compounds into hydrophilic film-forming matrices is often inefficient because many natural antioxidants are sensitive to oxygen, light, temperature, and pH and may also interact unfavorably with the polymer network during processing and storage, resulting in reduced activity and limited functional performance.^5,9^

In this context, emulsified systems have gained attention as alternatives for incorporating bioactive compounds into biopolymer-based films. Beyond acting as encapsulation systems, emulsions may also affect film morphology and, consequently, the optical, barrier, and mechanical properties. A recent review by Reis, Gomes, and do Amaral Sobral^6^ emphasized that emulsion characteristics such as droplet size, emulsion type, and emulsifier nature strongly influence the final properties of active films. Experimental studies have also shown that emulsified lipid phases can alter the internal organization of film matrices and improve barrier-related properties. For example, Zhao et al.^3^ reported that the incorporation of Pickering emulsion into potato starch/PVA film modified matrix structure and improved functional performance, while Tessaro et al.^9^ demonstrated that double emulsions containing pitanga leaf extract could be successfully incorporated into gelatin- and chitosan-based films, affecting antioxidant and barrier properties.

Among these systems, water-in-oil-in-water (W/O/W) double emulsions are particularly interesting because they provide a more complex interfacial structure, in which an internal aqueous phase is protected by an oil layer and dispersed in an external aqueous phase.^10,11^ This architecture is especially relevant for compounds extracted in polar media or highly sensitive to environmental degradation since it may reduce direct exposure of the active compounds to the external environment and facilitate their incorporation into hydrophilic polymer matrices. Although W/O/W emulsions have already been applied in food and pharmaceutical systems, their use in biopolymer-based films remains more limited than that of conventional emulsions and nanoemulsions, and their effects are strongly formulation-dependent.

This is particularly relevant for annatto-derived carotenoids, whose extraction has traditionally relied on organic solvents, such as ethanol, acetone, or hexane, raising environmental and safety concerns. In this context, natural deep eutectic solvents (NADESs) have emerged as promising green alternatives because they are composed of low-toxicity constituents and can efficiently extract bioactive compounds while preserving their chemical stability and antioxidant functionality.^8,12,13^ Recent work also highlights the potential of NADES not only as green extraction media but also as functional processing aids and additives in biopolymer film formation and coating systems, where they influence polymer mobility, film homogeneity, and functional performance.^14^ Although plant extracts have been widely incorporated into starch-based films, preserving the stability of bioactive compounds, particularly carotenoids, remains challenging. Moreover, relatively few studies have systematically evaluated or compared the retention of bioactivity after incorporation into the film matrices.

Despite the growing interest in NADES for extraction, much less attention has been given to how NADES-derived extracts behave when incorporated into structured delivery systems and subsequently into starch-based films. Therefore, the relevance of combining NADES extraction with W/O/W encapsulation lies not simply in associating two existing technologies but in addressing a specific formulation challenge: how to incorporate a polar extract containing sensitive antioxidant compounds into a hydrophilic starch matrix while minimizing degradation and improving functional performance. To the best of our knowledge, few studies have explored the combination of NADES-derived plant extracts, W/O/W double emulsions, and starch-based films.

Based on this gap, this study investigated the encapsulation of an NADES-based annatto seed extract in a W/O/W emulsion and its incorporation into potato starch-based films. The objective was to evaluate the physicochemical properties and stability of the emulsion, as well as its effects on the structural, physicochemical, mechanical, optical, barrier, and antioxidant properties of the resulting films. Starch-based films without added NADES–annatto seed extract and films containing the nonencapsulated extract were used as controls.

We hypothesize that the multilayer architecture of the W/O/W emulsion enhances the protection of NADES-derived bioactive compounds and improves the barrier properties of starch-based films. Specifically, the internal aqueous phase confines the extract, reducing direct exposure to environmental stressors, while the oil layer and multilayer droplet interfaces create tortuous pathways that limit water and light penetration, leading to improved water vapor permeability and ultraviolet–visible (UV–vis) barrier performance. This hypothesis can be tested through measurements of the antioxidant activity, water vapor permeability, and optical properties of the films.

## 2 Material and Methods

### 2.1 Material

For the preparation of the NADES, choline chloride and lactic acid were purchased from Sigma-Aldrich (St. Louis, MO, USA). Annatto seeds were purchased in bulk from a local supplier as a single batch and stored at −18 °C in the absence of light until extraction. For antioxidant analyses and quantifications, the following chemical reagents were used: (±)-6-Hydroxy-2,5,7,8-tetramethylchromane-2-carboxylic acid (Trolox), 2,2′-Azino-bis­(3-ethylbenzothiazoline-6-sulfonic acid) (ABTS), absolute ethyl alcohol P.A. (Êxodo Científica, Sumaré, SP, Brazil), hexane P.A. (Synth, Diadema-SP, Brazil), and Nile Red (Sigma-Aldrich, St. Louis-MO, USA).

Potato starch (moisture content of 16% w/w and apparent amylose content of 22.6 g/100 g starch (dry basis)) used as a biopolymer for film production was provided by Zona Cerealista (São Paulo, SP, Brazil), and glycerol (95% purity; Synth, São Paulo, SP, Brazil) was employed as a plasticizer.

For the preparation of the W/O and W/O/W emulsions, commercial soybean oil (Cargill, Primavera do Leste, MT, Brazil) was used as the oil phase. Poly­(glycerol polyricinoleate) (Grinsted PGPR, DuPont, São Paulo, SP, Brazil), Tween 80 (Sigma-Aldrich, St. Louis, MO, USA), and sodium caseinate (>90% purity, Alibra Ingredients SA, Campinas, SP, Brazil) served as emulsifying agents.

### 2.2 Production of NADES-Based Annatto Seed Extract

NADES was prepared following the procedure described by Balabram et al.^8^ by combining choline chloride and lactic acid (CC/LA) in a 1:1 molar ratio, corresponding to 80% (w/w), with the addition of 20% (w/w) distilled water. The mixture was subjected to an ultrasonic bath (LT-210, LimaTec, São Carlos-SP, Brazil) at 50 °C for 45 min. The resulting CC/LA was stored at 25 °C until further use. The resulting NADES was characterized in terms of pH (1.28 ± 0.02), density (1.145 ± 0.007 g cm^–3^), viscosity (19.4 ± 0.3 mPa s), and polarity, which was estimated indirectly via the transition energy (E_NR_ = 48.1 ± 0.1 kcal mol^–1^), of Nile Red dye, and stored at 25 °C until further use.^8^

NADES-based annatto seed extract (NE) was obtained via ultrasound-assisted extraction, following the procedure described by Balabram et al.^8^ Briefly, 1 g of annatto seeds was combined with 15 mL of CC/LA and subjected to an ultrasonic bath at 50 °C for 45 min. After extraction, the mixture was filtered through a paper filter (80 g/m^2^), and the resulting NE was stored protected from light and used immediately. This NE was previously characterized in terms of its physicochemical and active properties in a study by Balabram et al.^8^

### 2.3 Preparation of W/O/W Emulsion Loaded with NADES-Based Annatto Seed Extract

The W/O/W emulsion was prepared in two sequential steps, based on Tessaro, Luciano et al.,^9^ with slight modifications. In the first step, a primary water-in-oil (W/O) emulsion was obtained using the NE as the internal aqueous phase, corresponding to 20% (w/w) of the total formulation. The oil phase (80%, w/w) consisted of soybean oil containing PGPR (3 g/100 g of soybean oil). The previously prepared NE was added dropwise to the oil phase under high-shear mixing using a rotor–stator homogenizer (Ultraturrax IKA T25, Labotechnik, Germany) operating at 15,000 rpm for 5 min. The resulting coarse W/O emulsion was then further homogenized by an ultrasonic probe sonicator (QR 850W, Ultronique-Ecosonics, Indaiatuba, SP, Brazil) at 20 kHz and 80% amplitude, applying three 30 s cycles while keeping the system in an ice bath.

In the second step, the W/O/W emulsion was produced by dispersing the W/O emulsion (30%, w/w) into the external aqueous phase (70%, w/w). The outer water phase was prepared with Tween 80 (3 g/100 g of water) and sodium caseinate (0.5 g/100 g of water). The W/O emulsion was added dropwise to the external phase under magnetic stirring (1000 rpm for 5 min) to form an initial coarse W/O/W emulsion. This system was subsequently processed using an ultrasonic probe sonicator equipped with a 13 mm macrotip probe (nominal power of 850 W, 20 kHz frequency, 80% amplitude) for four cycles of 30 s, maintaining the system in an ice bath to minimize heating. Under these conditions, a W/O/W emulsion was obtained, in which NE-loaded W/O droplets were dispersed within the continuous aqueous phase. The freshly prepared W/O/W emulsion was immediately characterized and then used for the starch-based film production.

### 2.4 Characterization of W/O/W Emulsion Loaded with NADES-Based Annatto Seed Extract

#### 2.4.1 pH and Color

The pH of the W/O emulsion was estimated using universal indicator paper, whereas the pH of the W/O/W emulsion was measured using a portable pH meter (K39–220, Kasvi, Pinhais, SP, Brazil). Given the complex and heterogeneous nature of the emulsified systems, this method provided approximate values intended for comparative analysis. The color parameters (L*, a*, and b*) of both emulsions were assessed using a colorimeter (Delta Vista d.8, Delta Color, São Leopoldo, RS, Brazil), operating in reflectance mode with the CIELab scale, illuminant D65, a 10° observation angle, and a measuring opening of 8 mm. The samples were placed in transparent containers, ensuring that the measurement window was completely covered by the emulsion. From the a* and b* values, the hue angle (h*) and chroma (C*) were also obtained by the equipment, according to eqs 1 and 2, respectively.

1 $$h^{*}=\arctan\left(\frac{b^{*}}{a^{*}}\right)$$

2 $$C^{*}=\sqrt{{\left(a^{*}\right)}^{2}+{\left(b^{*}\right)}^{2}}$$

#### 2.4.2 Creaming Stability and Encapsulation Efficiency

The physical stability of the emulsions was evaluated by determining the creaming index (CI), according to Hosseini et al.,^15^ with slight modifications. Approximately 5 mL of the freshly prepared emulsion was transferred into Eppendorf tubes, which were sealed and stored at 4 °C for up to 14 days. The emulsions were visually evaluated at day 0, day 7 and day 14 by photographic recording to monitor possible phase separation. The total height of the emulsion column (*H*_T_) and the height of any separated serum or cream layer (*H*_C_) were determined by visual inspection of the tubes. These parameters were used to calculate the creaming index according to eq 3.

3 $$\mathrm{CI}=\frac{H_{C}}{H_{T}}$$

The encapsulation efficiency (EE) of annatto seed extract in the emulsions was evaluated based on solvent extraction, as described by Santos et al.^16^ Briefly, 1 g of sample was mixed with an ethanol/n-hexane solution (2:3, v/v) and vortexed to extract the nonencapsulated fraction. After phase separation, the organic phase was collected, and the extraction procedure was repeated twice. The combined organic phases were analyzed using a UV–vis spectrophotometer (UV-M51, BEL Engineering, Monza, MB, Italy) at 450 nm using a calibration curve prepared with annatto seed extract. The encapsulation efficiency was calculated as the difference between the theoretical content (TC), corresponding to the absorbance of the nonencapsulated annatto seed extract (reference sample measured under identical conditions), and the recovered content (RC) obtained from the emulsions, according to eq 4.

4 $$\mathrm{EE}\;\left(\%\right)=\frac{\mathrm{TC}-\mathrm{RC}}{\mathrm{TC}}\times 100$$

#### 2.4.3 Droplet Size, Polydispersity Index (PDI), and ζ-Potential Measurement

Droplet size (indicated by mean hydrodynamic diameter), polydispersity index (PDI) of the W/O and W/O/W emulsions, and ζ-potential of the W/O/W emulsion, were determined by photon correlation spectroscopy using a Zetasizer Nano Instrument (ZS90, Malvern Instruments, Malvern, U.K.) with a detectable size range of 0.3 nm to 10 μm according to Tessaro et al.^9^ and Tessaro et al.^17^

For droplet size and PDI measurements, W/O emulsion was diluted 1:100 in chloroform, while W/O/W emulsion was diluted 1:800 in distilled water. Measurements were conducted at 25 °C using a He–Ne laser (λ = 627 nm) at a 90° scattering angle. ζ-Potential was determined at the same instrument equipped with a ZEN 1020 dip cell, at 25 °C. Before analysis, W/O/W emulsion was diluted 1:800 in distilled water to obtain an appropriate particle concentration for electrophoretic measurements. Each sample was analyzed in triplicate, and the ζ-potential values (mV) were automatically calculated by instrument software based on the electrophoretic mobility of the droplets.

#### 2.4.4 Microstructure by Fluorescence Microscopy

The microstructure of the W/O and W/O/W emulsions was observed by fluorescence microscopy, following the procedure described by Tessaro, Gonçalves, et al.,^18^ with minor modifications. Samples were examined using a digital fluorescence microscope (Mateo FL, Leica Microsystems, Wetzlar, Hesse, Germany) equipped with a 60× objective. The oil phase was labeled with Nile Red prepared in ethanol (0.25 mg/mL) and mixed with the emulsions at a 1:10 (v/v) dye-to-sample ratio.

#### 2.4.5 Rheological Behavior

The rheological behavior of the W/O and W/O/W emulsions was evaluated using a controlled-stress rheometer (AR-1000N, TA Instruments, New Castle, DE, USA) equipped with a Peltier system for temperature control, following the procedure described by Costa et al.^19^ Steady shear flow measurements were carried out at 25 °C using a cone–plate geometry (40 mm diameter, 2° cone angle, 55 μm gap). The apparent viscosity was recorded over a shear-rate range from 0.1 to 350 s^–1^, performing three consecutive up–down–up ramps for each sample.

#### 2.4.6 Antioxidant Activity

The antioxidant activity of the W/O and W/O/W emulsions was determined by the ABTS and DPPH radical-scavenging assays. Before analysis, 0.5 g of each emulsion was transferred to test tubes containing 3.0 mL of ethanol and 2.0 mL of hexane, according to Reis et al.,^20^ with modifications. The tubes were vortexed every 10 min for 20 min and then allowed to stand for phase separation. Both the ethanolic and hexane phases were collected, and the extraction procedure was repeated twice for the W/O emulsion and three times for the W/O/W emulsion. The combined extracts were used in the antioxidant assays to minimize losses associated with the partitioning of antioxidant compounds between solvents. Solvent blanks (ethanol and hexane without emulsion) were measured to correct for background absorbance. For the ABTS assay, antioxidant activity was determined according to Re et al.^21^ Trolox calibration solutions (0.02–0.38 mg/mL) were prepared to construct a standard curve (*R* ^2^ = 0.996), as reported by Balabram et al.^8^ For the DPPH assay, antioxidant activity was determined according to Maniglia et al.^22^ Trolox calibration solutions (0.001–0.252 mg/mL) were prepared to construct a standard curve (*R* ^2^ = 0.999). The antioxidant activity of the emulsions was expressed as mg Trolox equivalents (TE)/g of emulsion and mg TE/g of NE.

### 2.5 Production of Starch-Based Films

Starch-based films were prepared using the casting method, based on a previous methodology by Maniglia et al.^22^ with some modifications. Four formulations were produced: a control starch-based film (C) containing only starch and glycerol; two starch-based films incorporating the NE at 5% and 10% relative to starch mass (NE-5% and NE-10%); and one starch-based film containing 5% of the NE in a W/O/W emulsion (DE-5%) (Table 1). In addition, two extra formulations were prepared only for preliminary visual evaluation: a starch-based film containing 10% of the NE in a W/O/W emulsion (DE-10%) and a starch-based film containing 5% of the NE in a W/O emulsion (SE-5%). Due to their oil separation, these formulations were not selected for further analyses, but their visual appearance was recorded and is discussed in the results section.

**Table 1:** Summary of Starch-Based Film Formulations and Analyses^a^

formulation	type of incorporation	concentration (%)	analyses included	notes
C	-	-	all quantitative analyses	-
NE-5%	free extract	5	all quantitative analyses	-
NE-10%	free extract	10	all quantitative analyses	-
DE-5%	W/O/W emulsion	5	all quantitative analyses	stable
DE-10%	W/O/W emulsion	10	visual appearance only	unstable
SE-5%	W/O emulsion	5	visual appearance only	unstable

a Four main formulations (C, NE-5%, NE-10%, and DE-5%) were fully characterized using quantitative analyses. Two additional formulations (DE-10% and SE-5%) were prepared only for preliminary visual evaluation and were excluded from quantitative analyses due to instability.

Starch suspensions were prepared at a concentration of 5 g of starch/100 g of suspension (dry basis). The mixtures were heated in an ultrathermostatic bath (MA-184, Marconi, Piracicaba, SP, Brazil) at 85 °C under constant mechanical stirring at 350 rpm (RW20 digital, IKA, Campinas, SP, Brazil) for 30 min to promote starch gelatinization. For the C film, glycerol, used as a plasticizer, was added at 25 g/100 g of starch, and the suspension was kept under heating for an additional 15 min. Then, NE, W/O or W/O/W emulsions were incorporated into the starch suspension and stirred at 350 rpm for 10 min at 40 °C to ensure complete homogenization.

The resulting film-forming solutions were poured into 12 cm × 12 cm plates, using approximately 25 g of the gelatinized suspension per plate (corresponding to the experimentally defined casting volume). The film-forming solutions were dried in a forced-air oven (MA035/5, Marconi, Piracicaba, SP, Brazil) at 30 °C for 24 h. Once dried, the starch-based films were conditioned for 48 h at 25 °C and 58% relative humidity, using a saturated NaBr solution, before analysis. For scanning electron microscopy and Fourier transform infrared spectroscopy, the samples were conditioned over silica gel prior to testing to minimize moisture interference.

### 2.6 Characterization of Starch-Based Films

#### 2.6.1 Thickness, Visual Appearance, Opacity, Color, and UV/vis Radiation Barrier

The film thickness was measured to the nearest 0.001 mm at 5 random locations on the surface using a digital micrometer (Mitutoyo Corp., Sakado, Japan). The average thickness was calculated from the measurements.

The visual appearance of the films was evaluated by analyzing photographs using an iPhone 13 camera (Apple Inc., Cupertino, CA, USA) to assess their uniformity and homogeneity. Photographs of the films were taken under controlled lighting conditions, allowing for a visual inspection of color consistency and texture uniformity across the film surfaces.

The opacity was determined using rectangular film samples (12 mm × 40 mm), which were positioned in a UV–Vis spectrophotometer (UV-M51, BEL Engineering, Monza, MB, Italy) so that light passed completely through the films during measurement. The thickness of each film was measured at three random positions using a digital micrometer (Mitutoyo Corp., Sakado, Japan), and the average thickness was calculated. The absorbance of each film at 600 nm (*A*_600_) was recorded, and opacity was calculated according to eq 5, where *x* is the average film thickness (mm).^23^

5 $$\mathrm{opacity}=\frac{A_{600}}{x}$$

The color parameters of the films were assessed using a colorimeter (Delta Vista d.8, Delta Color, São Leopoldo, RS, Brazil), operating in reflectance mode with the CIELab scale, illuminant D65, a 10° observation angle, and a measuring opening of 16 mm. The films were placed on a standard white plate to ensure consistent measurement conditions. The total color difference (ΔE*) was calculated according to eq 6, where ΔL* = L*_sample_ – L*_standard_ (93.43), Δa* = a*_sample_ – a*_standard_ (−0.37), and Δb* = b*_sample_ – b*_standard_ (0.50).

6 $${\Delta E}^{*}=\sqrt{{\left({\Delta L}^{*}\right)}^{2}+{\left({\Delta a}^{*}\right)}^{2}+{\left({\Delta b}^{*}\right)}^{2}}$$

The UV/vis radiation barrier properties of the films were evaluated by measuring transmittance over the wavelength range of 200 to 800 nm using a UV–vis spectrophotometer (UV-M51, BEL Engineering, Monza, MB, Italy). Rectangular film samples (12 mm × 40 mm) were prepared and placed in the cuvette such that light passed through the films during the measurement.

#### 2.6.2 Moisture Content, Wettability, Solubility in Water, and Water Vapor Permeability (WVP)

Film samples with known initial weights were dried in a forced-air circulation oven at 60 °C for 48 h to determine their moisture content. The moisture content was calculated as the g of water/100 g of wet film. For the solubility test, film samples (2 cm diameter and known weight) were immersed in 50 mL of distilled water and shaken at 25 rpm and 25 °C for 24 h using an orbital shaker (MA141, Marconi, Piracicaba, SP, Brazil). After this period, the insoluble part of the films was separated by filtration and dried at 60 °C for 48 h in the forced-air circulation oven.

The wettability was measured by water contact angle (WCA) of the films’ air-side surfaces using an optical tensiometer (Attention Theta Lite, Biolin Scientific, Sweden), equipped with advanced image analysis software. The film samples were securely mounted on the instrument support, and a droplet of Milli-Q water was carefully placed on the surface using a precision syringe, according to Tessaro, Luciano, et al.,^9^ with modifications. The contact angle images were recorded at 0, 10, and 60 s of exposure, and the WCA values were extracted from the images at 10 s.

The solubility in water was calculated as the difference between the initial and final weights of the films, based on dry mass.^9^ Both solubility and moisture content tests were performed in triplicate to ensure accuracy and reproducibility of the results.

WVP of the films was determined at 25 °C, following a modified version of the ASTM E96–80 standard method^24^ from Tessaro, Luciano, et al.^9^ Round film samples (30 mm diameter) were sealed over the circular openings of permeation cells containing silica gel, and the cells were placed in desiccators containing distilled water. After sealing the films, the cells were weighed every hour for 7 h to track the weight gain. The WVP values of the films were calculated according to eq 7, where Δ*g*/Δ*t* is the rate of weight change (g/h), *x*, is the average film thickness (mm), *A*, is the film permeation area (0.00196 m^2^), and Δ*P*, is the partial pressure difference (3.168 kPa).

7 $$WVP\;(g\;mm/m^{2}\;h\;kPa)=\frac{\Delta g}{\Delta t}\left(\frac{x}{A\Delta P}\right)$$

#### 2.6.3 Functional Groups by Fourier Transform Infrared (FTIR)

Fourier transform infrared (FTIR) spectra were obtained from the Fourier transform infrared spectrophotometer (IRAffinity-1, Shimadzu Corporation, Kyoto, Japan), equipped with an Attenuated Total Reflectance (ATR). Analyses were performed from 4000 to 600 cm^–1^.

#### 2.6.4 Morphology, Crystallinity, Thermal, and Mechanical Properties

The morphological characteristics of the film surface and cross-section were examined by scanning electron microscopy (SEM) on a scanning electron microscope (LEO 440, LEO Electron Microscopy Ltd., Cambridge, U.K.), operated at 15 kV. For the cross-sectional images, the samples were frozen with liquid nitrogen and fractured to obtain a straight cross section. All samples were placed on an aluminum specimen stub and coated with gold before the analysis.

The film samples were analyzed using an X-ray diffractometer (D8 Advance, Bruker AXS GmbH, Karlsruhe, Germany) equipped with a position-sensitive detector (LynxEye, Bruker AXS GmbH, Karlsruhe, Germany) and a Cu Kα radiation source (λ = 1.5418 Å). The data were obtained in the coupled Theta/2Theta mode with a scanning increment of 0.02 degrees and an irradiation time of 0.5 s per step, with 2θ angles varying between 3° and 60°. The crystallinity index of the materials was obtained through the software DIFFRAC EVA (v6.1, Bruker AXS GmbH, Karlsruhe, Germany).

The thermal properties of the films were evaluated using a differential scanning calorimeter (DSC, Q100, TA Instruments, New Castle, DE, USA). Samples were placed in hermetically sealed aluminum pans and heated from −70 to 150 °C at 10 °C/min in an inert atmosphere (50 mL/min N_2_). The glass transition temperature, melting temperatures, and melting enthalpies were calculated directly from the thermal curves using the equipment software.

The mechanical properties of the films were assessed through axial tension tests following the ASTM D882-12 standard.^25^ The analysis was carried out with a texturometer (TA.XTplusC, Stable Micro Systems Ltd., Godalming-Surrey, U.K.) with a load cell of 50 kgf (490 N). The film samples, cut into 15 mm × 100 mm strips, were mounted in the grips of the instrument, which were separated by 50 mm. The test was conducted at a crosshead speed of 1 mm/s. The tensile strength and elongation at break were derived directly from the stress–strain curve, while the elastic modulus was calculated from the slope of the linear region of the curve. Data analysis was performed using the Texture Expert V.1.22 software (SMS).

#### 2.6.5 Antioxidant Activity

The antioxidant activity of the films was evaluated using the ABTS and DPPH methods, as described in Section 2.4.6. The results were expressed as mg TE/g of NE. Prior to analysis, 500 mg of the sample were mixed with 5 mL of methanol and stirred for 3 h at room temperature (approximately 25 °C) in a dark environment, following a modified procedure described by Maniglia et al.^22^ After the extraction process, the supernatant was collected for further analysis. The extract obtained from the control film was used as a matrix blank, and its antioxidant activity was discounted from the values obtained for the active films.

### 2.7 Statistical Analysis

Unless otherwise stated, all measurements were performed in triplicate using independently prepared samples, and results are expressed as mean ± standard deviation. Statistical treatment of the data was carried out using Statistica software (version 7, StatSoft Inc., Hamburg, Germany). Differences among means were assessed by one-way analysis of variance (ANOVA), and Tukey’s multiple comparison test was applied to identify significant differences at a significance level of α = 0.05.

## 3 Results and Discussion

### 3.1 Characterization of W/O and W/O/W Emulsions

#### 3.1.1 pH and Color

The pH values obtained for the W/O and W/O/W emulsions confirm the presence of the acidic NADES-based annatto extract within both systems (Table 2). The W/O emulsion exhibited an estimated pH close to 2, whereas the W/O/W emulsion presented a pH of 1.77 ± 0.01. It is important to note that the pH of the W/O emulsion was estimated using indicator paper due to the oil-continuous nature of the system, while the pH of the W/O/W emulsion was determined using a calibrated pH meter. Therefore, direct quantitative comparisons between the two values should be interpreted with caution. Nevertheless, both emulsions exhibited strongly acidic characteristics, reflecting the incorporation of the NADES-derived annatto extract into the formulations.^26^ To the best of our knowledge, studies specifically addressing emulsions encapsulating NADES-based extracts remain extremely limited.

**Table 2:** pH, Color Parameters, Creaming Index, Encapsulation Efficiency, and Antioxidant Activity of W/O and W/O/W Emulsions, and ζ-Potential of the W/O/W Emulsion^a^,^b^

properties	W/O emulsion	W/O/W emulsion
pH	∼2	1.77 ± 0.01
L*	41.0 ± 0.3b	80.8 ± 0.1a
a*	3.1 ± 0.1a	0.7 ± 0.0b
b*	20.9 ± 0.2a	9.3 ± 0.1b
C*	21.2 ± 0.2a	9.3 ± 0.1b
h*	81.6 ± 0.4b	85.5 ± 0.2a
creaming index (%)	0	0
encapsulation efficiency (%)	35.9 ± 1.6b	95.5 ± 0.5a
ζ-potential (mV)	-	–26.55 ± 1.09
ABTS (mg TE/g emulsion)	1.18 ± 0.10a	0.30 ± 0.01b
ABTS (mg TE/g annatto seed)	5.88 ± 0.52a	4.92 ± 0.09b
DPPH (mg TE/g emulsion)	2.72 ± 0.12a	0.43 ± 0.09b
DPPH (mg TE/g annatto seed)	13.60 ± 0.55a	7.17 ± 1.50b

a Different letters on the same line indicate significant differences between W/O and W/O/W according to Tukey’s test (*p* < 0.05). TE: Trolox equivalent.

b Means ± standard deviation (*n* = 3).

Visually, the W/O emulsion exhibited a light orange appearance, whereas the W/O/W emulsion was predominantly white and opaque (Figure 1a). These visual differences were quantitatively supported by the colorimetric parameters (Table 2). The W/O/W emulsion showed a significantly higher L* value, indicating a much lighter system, consistent with the strong light scattering characteristic of W/O/W emulsions containing multiple interfaces.^10^ Both samples showed positive a* and b* values, but the W/O emulsion exhibited higher intensities (*p* < 0.05), confirming its more pronounced orange coloration. Similar observations have been reported for oil-continuous emulsions containing lipophilic pigments, in which enhanced solubilization and dispersion intensify red–yellow chromaticity.^27^

**Figure 1:**
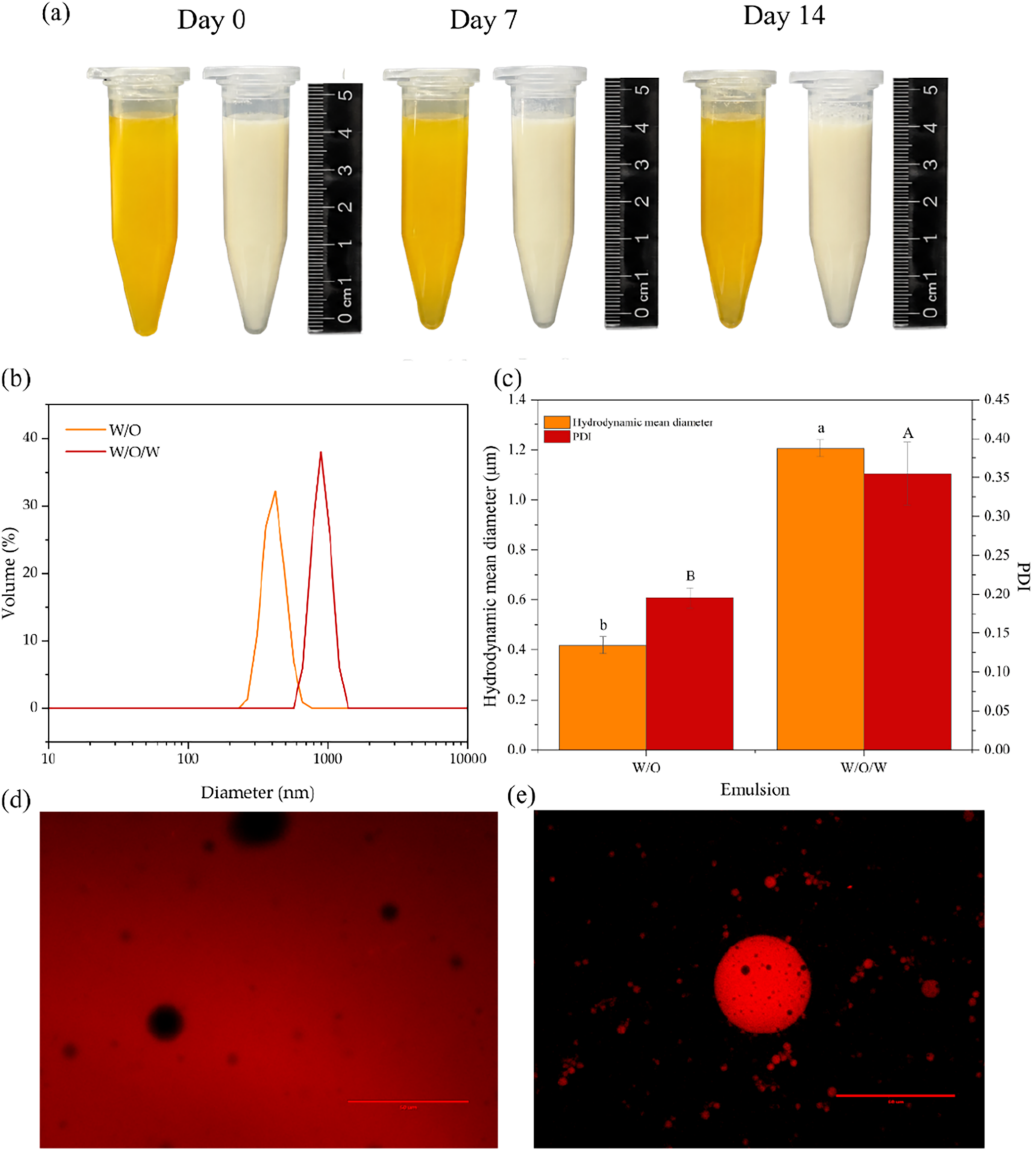
(a) Visual appearance of the W/O emulsion (left) and W/O/W emulsion (right) during 14 days of storage (Day 0, Day 7, and Day 14), (b) Droplet size distribution, (c) hydrodynamic mean diameter and polydispersity index (PDI), and fluorescence microscopy images of (d) W/O and (e) W/O/W emulsions (scale bar is 50 μm). Different letters indicate significant differences between W/O and W/O/W emulsions according to Tukey’s test (*p* **<** 0.05).

Differences in C* further emphasize this effect (Table 2). The W/O emulsion presented a more saturated color, while the W/O/W system appeared less vivid. The reduced C* values observed in the W/O/W emulsion may be related to its more complex internal structure, since W/O/W systems contain multiple interfaces that can influence droplet stability and the release behavior of encapsulated compounds.^28^ The h* indicates that both emulsions lie in the yellow–orange region, but the W/O/W emulsion shifts toward a less reddish tone than the W/O, consistent with reduced pigment availability.^28^

Overall, the pH and color results suggest that the W/O/W system may modulate pigment availability and optical behavior by redistributing the NADES-based extract within the film matrix, which is expected to influence its stability and performance in subsequent film applications.

#### 3.1.2 Creaming Index and Encapsulation Efficiency

The physical stability of the emulsions was evaluated through the creaming index (CI), which is used as an indicator of gravitational phase separation in emulsified systems.^11,29,30^ The absence of cream or serum layer formation was confirmed by visual inspection and photographic documentation (Figure 1a), comparing samples at day 0, day 7, and day 14, which shows the maintenance of a homogeneous system over time. Both W/O and W/O/W emulsions exhibited no visible phase separation during the 14-day storage period. As a result, the creaming index was determined to be 0% for all of the samples. This behavior indicates high physical stability of the emulsions, likely associated with effective interfacial stabilization provided by the emulsifier system and the small droplet size distribution.^11^ These results agree with previous studies reporting that stable emulsions with strong interfacial layers and adequate electrostatic or steric stabilization can prevent gravitational separation phenomena such as creaming or sedimentation.^29^

The apparent encapsulation efficiency (EE) of annatto seed extract was significantly influenced by the emulsion architecture. The W/O emulsion exhibited an EE of 35.92 ± 1.48%, indicating moderate retention of bioactive compounds within the oil phase. This result suggests partial accessibility of the encapsulated compounds to solvent extraction, reflecting limited interfacial protection in the primary emulsification system.

In contrast, the W/O/W double emulsion showed a markedly higher EE, demonstrating the strong retention of annatto seed bioactive compounds within the internal phase. This improvement can be attributed to the presence of an additional aqueous layer, which acts as a physical barrier that reduces mass transfer and limits solvent penetration. The multilayered interfacial structure of double emulsions is widely recognized for enhancing the protection of lipophilic compounds by decreasing their extractability.^11,30^

The obtained EE values for the double emulsion are within the range reported for structured delivery systems containing carotenoids. Similar encapsulation efficiencies have been described for carotenoid-loaded emulsions and related systems, highlighting the susceptibility of these compounds to oxidation and degradation when not effectively protected.^16^ Moreover, improved retention in structured systems has been reported when compared to simpler formulations, reinforcing the role of emulsion architecture in bioactive protection.^29,30^

#### 3.1.3 Droplet Size, PDI, and ζ-Potential Measurement

The droplet size characterization revealed that both the W/O and W/O/W emulsions exhibited monomodal distributions (Figure 1b), indicating that the homogenization conditions and emulsifier concentrations adopted in this study were sufficient to generate relatively well-defined droplet populations. In the case of the primary W/O emulsion, monomodal distributions are commonly reported for systems stabilized with PGPR, because the high viscosity of the oil phase, combined with the strong interfacial activity of this emulsifier, favors the formation and stabilization of relatively uniform droplets.^9,31^ This point is particularly relevant, because adequate stabilization of the primary W/O emulsion is a prerequisite for the successful formation of the subsequent W/O/W system. In double emulsions, any instability in the primary interface may compromise the integrity of the internal aqueous droplets during the second-emulsification step.

For the W/O/W emulsion, the monomodal distribution also suggests that, under the selected formulation conditions, the combination of the internal phase fraction, surfactant concentrations, and homogenization procedure was sufficient to avoid extensive recoalescence or the formation of clearly distinct droplet populations. This behavior is not always observed for double emulsions, since bimodal or trimodal profiles are often reported for W/O/W systems containing plant extracts or bioactive compounds due to their greater structural complexity and the higher probability of droplet disruption during the second emulsification step.^18,32^ On the other hand, monomodal W/O/W distributions have also been reported in more stable formulations, such as systems coencapsulating vitamin C and β-carotene, in which emulsifier composition and phase organization played an important role in maintaining droplet uniformity.^33^ Therefore, the monomodal profile observed here suggests that the selected formulation provided adequate short-term structural organization for both emulsions.

The hydrodynamic mean diameter of the W/O emulsion was smaller than that of the W/O/W emulsion (*p* < 0.05), which aligns with the typical increase in droplet size when a second aqueous phase is entrapped within oil droplets (Figure 1c). W/O emulsions often exhibit smaller droplet size because only a single interfacial layer must be stabilized; systems prepared with PGPR typically show mean droplet diameters in the submicrometric to low-micrometric range, often between 0.3 and 1.5 μm, depending on formulation and internal water content.^17,32^ In contrast, W/O/W emulsions require stabilization of multiple interfaces and therefore tend to form larger droplets, generally ranging from 3 to 6 μm.^17^ Thus, the values obtained here fall within the expected range for stable food-grade W/O and W/O/W emulsions. Similar droplet sizes have been reported for W/O systems encapsulating *Eugenia uniflora* (pitanga) leaf extract, which presented droplet diameters on the order of 400 nm.^17^ Likewise, micrometer-scale droplets have also been described for W/O/W emulsions containing rosemary polyphenolic extract. The reported particle-size parameters place the droplets in the low-micrometer range, consistent with hydrodynamic diameters of approximately 1.0–1.5 μm, depending on formulation and on the specific size metric reported.^34^ The PDI values further support these observations (Figure 1c): the W/O emulsion showed a lower PDI (*p* < 0.05), consistent with a relatively narrow size distribution, whereas the W/O/W emulsion presented a higher PDI, reflecting its more complex internal structure and broader droplet size distribution.^10^ This behavior is expected because double emulsions are intrinsically more complex systems, in which variations in droplet breakup, secondary emulsification, and partial coalescence may broaden the size distribution.^10,32^ Even so, the fact that the W/O/W emulsion remained monomodal despite its higher PDI is an important indication that the system did not undergo marked phase separation or severe destabilization immediately after preparation. In addition, it is plausible that NADES components and associated bioactive compounds contributed to interfacial stabilization by reducing interfacial tension and delaying coalescence, as reported for similar systems containing polar bioactive extracts.^35,36^

The fluorescence microscopy images corroborated the droplet size results (Figure 1d,e), showing well-defined, spherical droplets in both emulsions, with the W/O/W system presenting more prominent and larger droplets surrounded by smaller ones dispersed in the continuous aqueous phase, an arrangement characteristic of stable W/O/W emulsions.^10,17^ The smaller droplets observed throughout the field in both micrographs also support the measured PDI and reflect the coexistence of microscale and submicroscale populations typically expected in food emulsions.^10^

Moreover, the ζ-potential (Table 2) measured for the W/O/W emulsion indicated moderate electrostatic stability.^11^ Although absolute ζ-potential values higher than 30 mV are commonly associated with highly stable dispersions, double emulsions may remain stable at lower values when additional steric stabilization mechanisms are present. In the present system, this is particularly plausible because the external aqueous phase contained Tween 80 and sodium caseinate, which likely contributed to interfacial protection not only by electrostatic repulsion but also by steric hindrance. Similar interpretations have been reported for emulsified systems stabilized by combinations of proteins and low-molecular-weight surfactants, in which ζ-potential alone does not fully explain the observed stability.^3,37^

Taken together, droplet size distribution, PDI, microscopy, and ζ-potential results indicate that the W/O/W emulsion was successfully formed and suggest short-term stability under the evaluated conditions, providing a structurally suitable system for incorporation into starch-based films.

#### 3.1.4 Rheological Behavior

Rheological analysis revealed clear structural differences among the emulsions (Figure 2), which can be directly associated with their distinct internal structures. The W/O emulsion showed nearly constant viscosity over the evaluated shear rate range, indicating Newtonian behavior under the tested conditions.^31^ This suggests that the dispersed internal aqueous droplets were sufficiently small and well-distributed within the continuous oil phase, without forming an extended droplet network capable of resisting flow.^13,30^ Such behavior has been reported for W/O emulsions stabilized with PGPR, in which the continuous lipid phase and relatively simple interfacial structure favor a more uniform flow response.^31^ Residual analysis (Figure S1A, Supporting Information) indicates that while the majority of residuals are close to zero, a slight systematic trend is observed, with larger deviations at lower shear rates. This pattern is consistent with what might be expected for complex emulsion systems and suggests that the Newtonian model generally captures the flow behavior without overinterpreting the fit.

**Figure 2:**
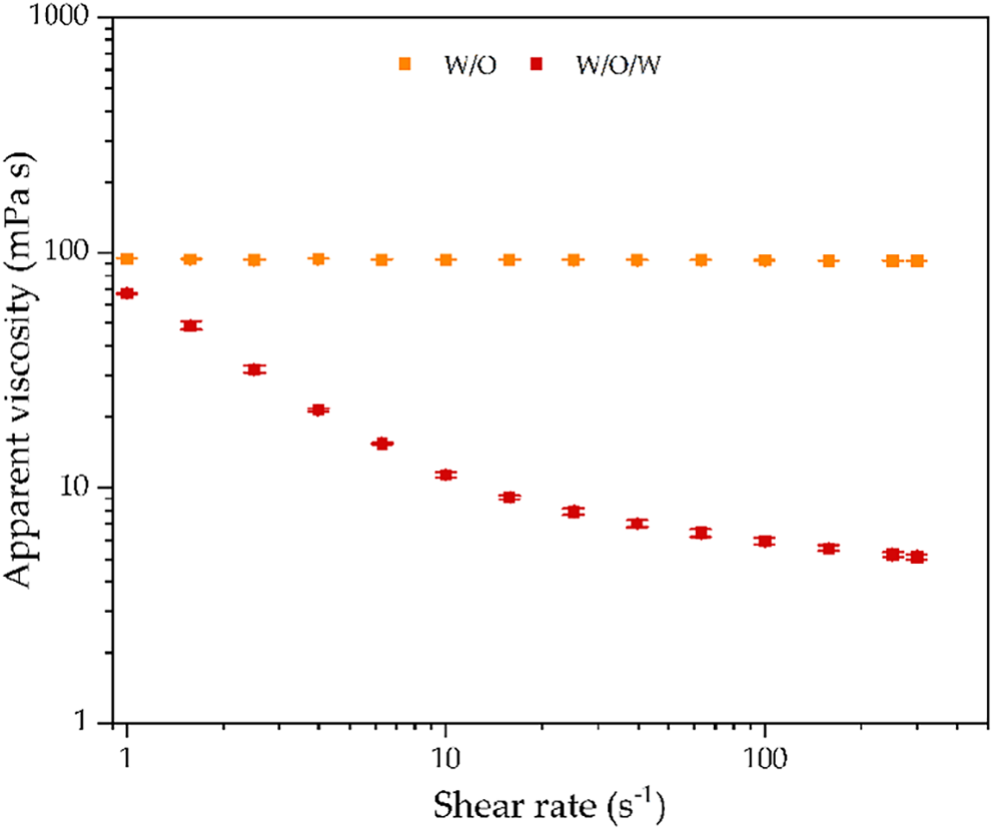
Rheological behavior (flow curve) of the W/O and W/O/W emulsions.

In contrast, the W/O/W emulsion showed higher apparent viscosity at low shear rates and a progressive decrease in viscosity as shear rate increased, characterizing pseudoplastic behavior. This result is supported by the Power-Law parameters, since the flow index (*n* < 1) indicates shear-thinning behavior and the consistency coefficient (*K*) reflects a more structured system at rest (Table 3). This response is typical of double emulsions and reflects the presence of larger composite droplets, a broader size distribution, and more frequent droplet–droplet interactions in the external aqueous phase.^11,32^ As shear increases, deformation and rearrangement of the internal droplets reduce structural integrity, resulting in lower apparent viscosity. In the present formulation, the presence of Tween 80 and sodium caseinate in the external phase likely also contributed to weak interdroplet associations, increasing viscosity at low shear and favoring structure breakdown under flow.^11^ From a practical standpoint, this behavior is favorable for film production, since a higher viscosity at rest may help reduce phase separation before casting, while shear thinning facilitates spreading during processing.

**Table 3:** Newtonian Model Parameters and Apparent Viscosity of the W/O Emulsion and Power-Law Parameters and Apparent Viscosity of the W/O/W Emulsion^a^,^b^

rheological model	W/O emulsion	W/O/W emulsion
Newtonian		-
η (mPa·s)	92.41 ± 0.22	-
*R* ^2^	0.999	-
Power-Law		
*K* (Pa·s^n^)	-	0.014 ± 0.001
*n*	-	0.823 ± 0.012
*R* ^2^	-	0.997
η at 100 s^–1^ (mPa·s)	92.69 ± 0.26a	5.94 ± 0.14b

a Different letters on the same line indicate significant differences between apparent viscosity of W/O and W/O/W emulsions according to Tukey’s test (*p* < 0.05).

b Means ± standard deviation (*n* = 3).

Residual analysis for the W/O/W emulsion (Figure S1B, Supporting Information) indicates that deviations between predicted and experimental viscosities are largest at low shear rates and decrease with increasing shear rate. This trend suggests that the Power-Law model adequately represents the overall shear thinning behavior, although minor deviations occur at low shear rates. These residuals provide quantitative confirmation of the model fit across the tested range, complementing the flow curve analysis.

#### 3.1.5 Antioxidant Activity

Interestingly, when the antioxidant activity was expressed relative to the amount of NE incorporated, both emulsions exhibited a high preservation of the extract’s radical-scavenging capacity (Table 2). For the ABTS assay, the antioxidant activity of both emulsions was statistically similar to that previously reported for the free NE^8^ (4.99 ± 0.62 mg TE/g annatto seed), indicating that the emulsification process did not markedly compromise the bioactive compounds responsible for antioxidant activity. Consistent results were observed in the DPPH assay, in which both emulsions exhibited substantial antioxidant activity and retained the radical-scavenging capacity of the encapsulated compounds. The agreement between ABTS and DPPH results strengthens the conclusion that the antioxidant compounds remained active after emulsification and that the observed differences between emulsions are primarily related to differences in compound accessibility rather than degradation. Similar behavior has been reported for açaí berry extract nanoemulsions, in which emulsified systems showed higher apparent antioxidant activity and improved phenolic retention compared with the nonemulsified extract.^38^ For W/O/W systems, such behavior has also been widely reported by Tessaro, Gonçalves, et al.,^18^ who observed that pitanga leaf extract encapsulated in W/O/W emulsions maintained its antioxidant activity, although lower ABTS and DPPH values were obtained than in the corresponding simple emulsions.

For both ABTS and DPPH assays, the W/O emulsion exhibited significantly higher antioxidant activity than the W/O/W emulsion (*p* < 0.05). In addition, the W/O emulsion presented antioxidant activity comparable to or higher than that of the free extract. This result may be associated with the improved dispersion of antioxidant compounds in the emulsified system, which can increase their contact with the radical medium. In W/O systems, hydrophobic antioxidants may become more effectively distributed within the oil phase and at the oil–water interface, which can increase their apparent accessibility during the assay.^10,39^ In contrast, the somewhat lower antioxidant activity observed for the W/O/W emulsion in both assays may reflect the more complex multilayer structure of the droplets, which can limit immediate accessibility of part of the encapsulated compounds during extraction and reaction steps. A similar trend has been reported for curcumin emulsions and polyphenol-rich nanoemulsions, in which antioxidant compounds are preserved after emulsification but may exhibit lower or altered apparent activity than simple systems due to differences in dispersion, accessibility, and diffusional or partitioning limitations during analysis.^38,40^ Therefore, the lower measured antioxidant activity does not necessarily indicate degradation of the bioactive compounds but rather is a consequence of their confinement within the multilayer structure.

These findings demonstrate that emulsification, particularly in the W/O/W configuration, preserves the antioxidant potential of NADES–annatto extract while modulating its accessibility, which is a desirable feature for controlled antioxidant delivery in active packaging systems.

### 3.2 Characterization of Starch-Based Films

#### 3.2.1 Thickness, Visual Appearance, Opacity, Color, and UV/Vis Radiation Barrier

The thickness of the starch-based films varied according to the formulation (Table 4). The control film and DE-5% showed similar thickness values, whereas NE-5% and NE-10% were significantly thinner (*p* < 0.05). In contrast, no significant difference was observed between NE-5% and NE-10% (*p* > 0.05), which presented the lowest thickness values among all formulations. This reduction may be associated with a denser packing of polymer chains during drying, possibly resulting from interactions between the extract components and the starch matrix that could influence matrix expansion and solvent retention. These observations align with previous reports describing how direct incorporation of bioactive extracts can affect the microstructure and physical properties of polysaccharide-based films.^5,22^ In contrast, the thickness of DE-5% remained similar to that of the control, indicating that incorporation through the W/O/W emulsion did not promote the same degree of matrix compaction, likely because the dispersed droplets occupied volume within the film-forming system and limited chain packing during solvent evaporation, as similarly observed for starch/PVA films containing emulsified lipid phases.^3^

**Table 4:** Thickness, Opacity, Color Parameters, Physicochemical Properties, and Water Contact Angle of Starch-based Films without (C) and with NADES–based Annatto Seed Extract at 5% (NE-5%) and 10% (NE-10%) or W/O/W Emulsion at 5% (DE-5%)^a^,^b^

properties	C	NE-5%	NE-10%	DE-5%
thickness (mm)	0.102 ± 0.001a	0.082 ± 0.004b	0.081 ± 0.002b	0.097 ± 0.002a
opacity (mm^–1^)	1.86 ± 0.14c	4.57 ± 0.37b	5.22 ± 0.51ab	6.05 ± 0.24a
L*	57.93 ± 0.16a	56.92 ± 0.10b	57.04 ± 0.06b	54.82 ± 0.04c
a*	–0.87 ± 0.05c	–0.72 ± 0.05b	–1.10 ± 0.07d	0.41 ± 0.03a
b*	1.02 ± 0.01c	0.83 ± 0.02d	1.11 ± 0.05b	1.94 ± 0.02a
ΔE*	37.50 ± 0.16c	38.51 ± 0.10b	38.40 ± 0.06b	40.64 ± 0.04a
moisture content (%)	10.5 ± 0.6a	10.0 ± 0.3a	9.9 ± 0.7a	5.6 ± 0.6b
solubility in water (%)	21.8 ± 1.7a	5.7 ± 1.1b	7.6 ± 0.8b	7.6 ± 0.6b
WVP (g mm/m^2^ h kPa)	1.84 ± 0.41a	1.39 ± 0.27ab	1.02 ± 0.10b	0.88 ± 0.02b
water contact angle (°)	33 ± 2c	74 ± 5a	73 ± 3a	46 ± 1b

a Different letters on the same line indicate significant differences between C, NE-5%, NE-10% and DE-5% according to Tukey’s test (*p* < 0.05). WVP: water vapor permeability.

b Means ± standard deviation (*n* = 3).

After drying, the C film and those containing free NE (NE-5% and NE-10%) were transparent, homogeneous, and visually uniform, indicating a good film-forming ability (Figure 3). In contrast, DE-5% exhibited a more opaque and slightly whitish appearance, with reduced surface uniformity. This behavior is attributed to light scattering caused by the presence of dispersed oil droplets within the starch matrix, which generate microheterogeneities and interfaces with different refractive indices.^9^ Similar visual effects have been reported for gelatin- and chitosan-based films incorporating W/O/W emulsions loaded with plant extracts.^5^

**Figure 3:**
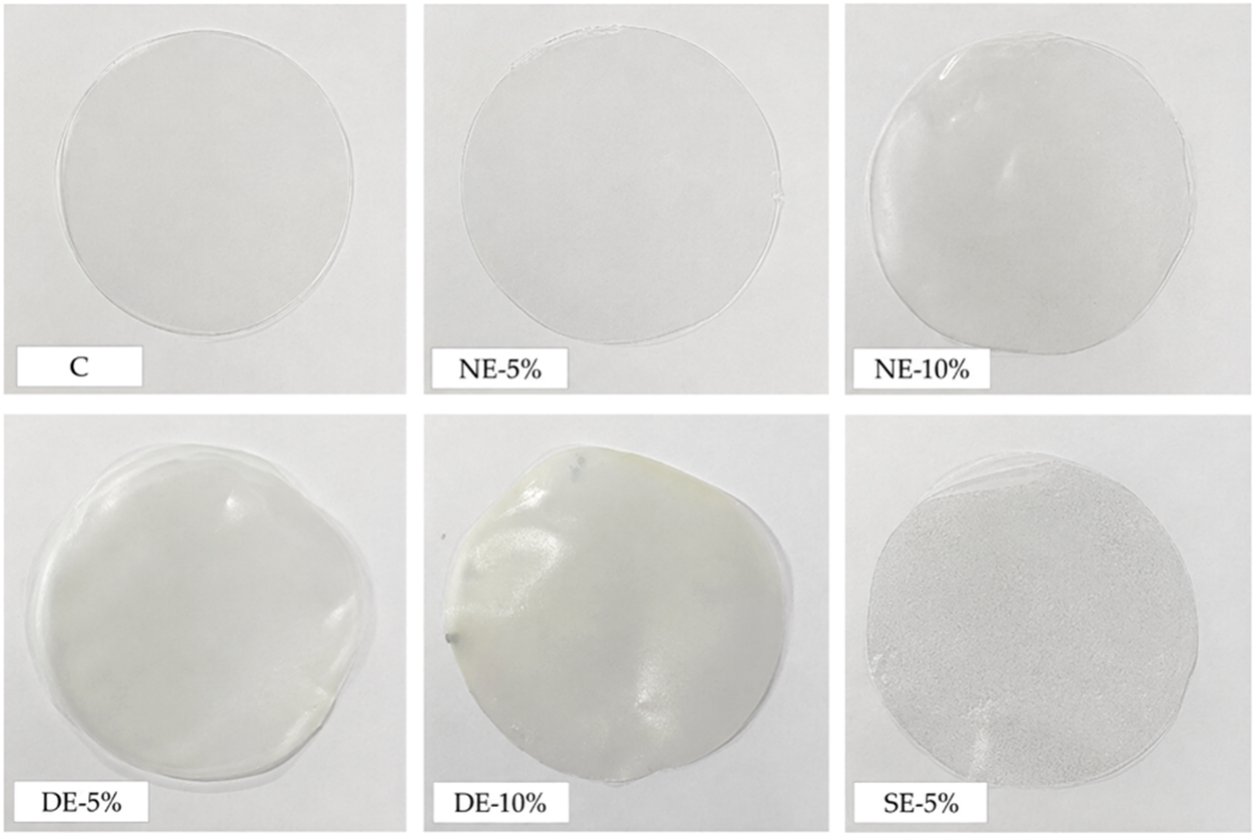
Visual appearance of starch-based films without (C) and with NADES–based annatto seed extract at 5% (NE-5%) and 10% (NE-10%), W/O/W emulsion at 5% (DE-5%) and 10% (DE-10%), or with W/O emulsion at 5% (SE-5%).

When the W/O/W emulsion concentration was increased to 10% (DE-10%), the films displayed pronounced turbidity, visible air bubbles, and regions of apparent phase separation, indicating emulsion destabilization during casting and drying. Likewise, films produced with the W/O emulsion (SE-5%) exhibited highly irregular and nonuniform surfaces, evidencing poor compatibility between the oil-continuous emulsion and the starch-based film-forming solution. These observations are consistent with previous studies reporting that polysaccharide matrices tolerate only limited amounts of dispersed lipid phases before phase separation occurs.^3,9^ Therefore, DE-10% and SE-5% were excluded from further characterization due to clear macroscopic instability (phase separation and structural heterogeneity), which compromised reproducibility and analytical reliability.

The opacity of the starch-based films varied, depending on the formulation (Table 4). Control films exhibited the highest transparency, whereas the incorporation of free NADES-based extract led to a noticeable increase in opacity, which appeared to be enhanced with higher extract concentrations. This trend may be associated with the presence of carotenoid pigments and potential microstructural heterogeneities induced during drying, which can influence light absorption and scattering.^6,8^ Films containing the W/O/W emulsion exhibited the greatest opacity among the studied formulations, likely due to the presence of dispersed oil droplets within the starch matrix, generating multiple internal interfaces and refractive index differences that can enhance light scattering.^6,9^

These observations align with previous reports showing that the inclusion of emulsified or oil phases generally increases film opacity and that droplet size and distribution can modulate this effect. Such microstructural features may contribute to the observed increase in opacity by creating additional light-scattering interfaces within the film.^40,41^ Despite the observed increases, all films can still be considered translucent, consistent with their potential application in packaging-colored foods where some light transmission is desirable.^20^

Color parameters (Table 4) corroborated the visual observations. The incorporation of free NE slightly affected the L*, a*, and b* values, whereas DE-5% showed a more pronounced color change, reflected by lower L* values and higher b* values, indicating increased yellowness. The higher total color difference (ΔE*) observed for DE-5% is associated with both the presence of carotenoid pigments and enhanced light scattering promoted by the emulsified system.^6^

The UV/vis radiation barrier properties of the films are shown in Figure 4a. Exposure to ultraviolet and visible light accelerates oxidative degradation and photo-oxidation of sensitive food components; therefore, improved radiation barrier properties are highly desirable for packaging materials.^43^ The incorporation of free NE significantly reduced transmittance in the UV region (200–400 nm) relative to the control film, and this effect was concentration-dependent, since NE-10% showed lower UV transmittance than NE-5%. This behavior is attributed to the presence of carotenoids and phenolic compounds, which act as chromophores and efficiently absorb UV radiation.^6,44^

**Figure 4:**
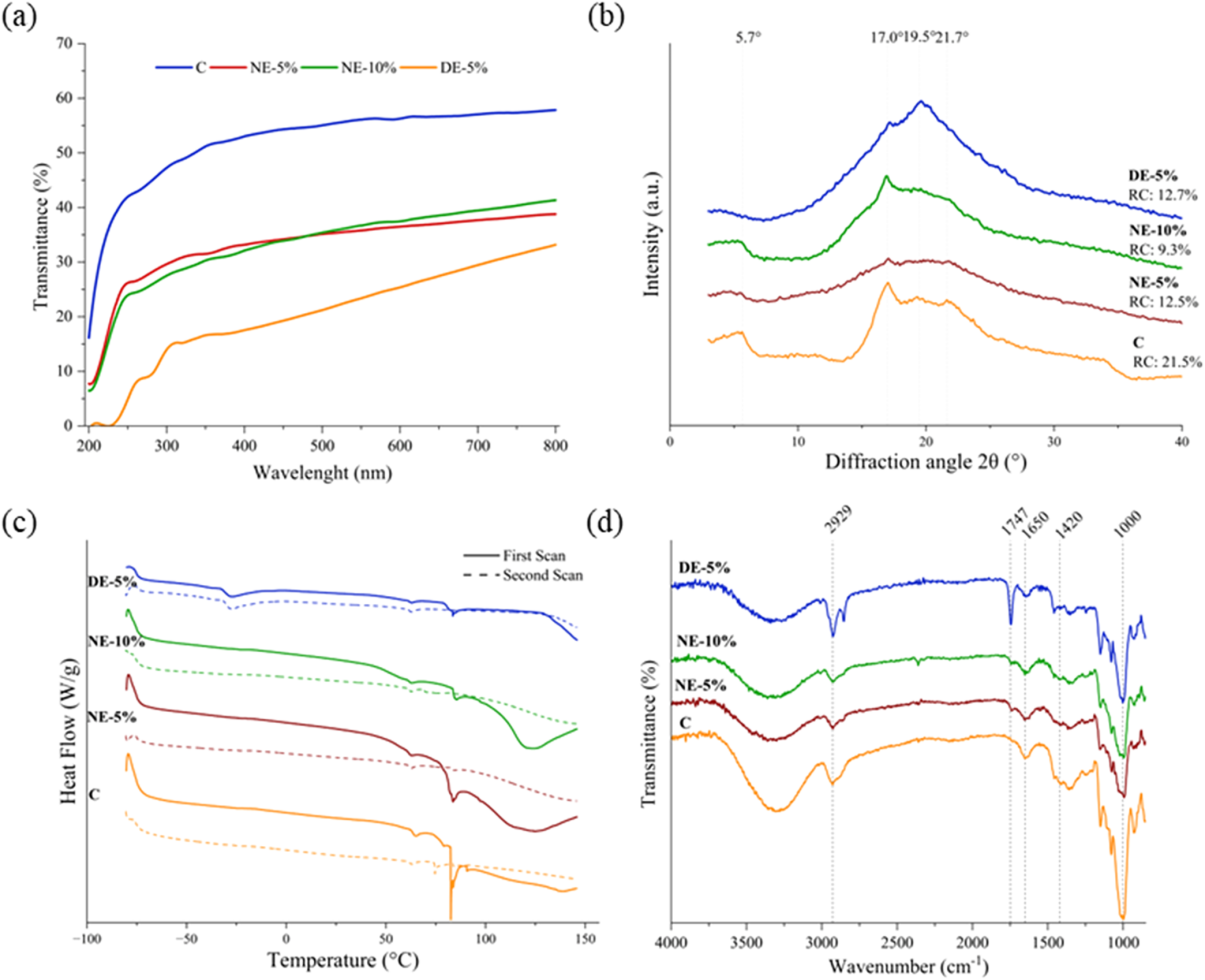
(a) UV/vis spectra, (b) X-ray diffractogram, (c) Thermal curve (Exo up), and (d) ATR-FTIR spectra of starch-based films without (C) and with NADES–based annatto seed extract at 5% (NE-5%) and 10% (NE-10%) or W/O/W emulsion at 5% (DE-5%).

The most pronounced reduction in transmittance across both UV and visible regions was observed for DE-5%. The dispersed oil droplets introduce controlled tortuosity within the polymer network, creating diffusion-resistant pathways that fundamentally alter moisture transport through the matrix. Multiphase systems containing emulsified lipid droplets are known to increase opacity and reduce transparency due to multiple internal interfaces, which scatter incident light and reduce its transmission through the material.^3,9^ Since film thickness differed among formulations, part of the variation in UV–Vis transmittance may be associated with thickness effects. However, the pronounced reduction in transmittance observed for DE-5% also suggests the contribution of structural factors related to the emulsion and the presence of carotenoids. According to Zhang et al.,^44^ films exhibiting UV transmittance values below 4% can be classified as effective UV-shielding materials. In the present study, films containing NE, particularly when incorporated via W/O/W emulsion, meet this criterion, demonstrating their potential application as active food-packaging materials with enhanced light-barrier properties. These findings demonstrate that W/O/W emulsions can act not only as carriers of bioactive compounds, but also as structural modifiers capable of influencing light–matter interactions and barrier behavior in polysaccharide-based matrices.

#### 3.2.2 Moisture Content, Wettability, Solubility in Water, and Water Vapor Permeability (WVP)

The moisture content of the films was not significantly affected by the incorporation of NE, since C, NE-5%, and NE-10% showed similar values (*p* > 0.05) (Table 4). This result suggests that, at the tested levels, the direct addition of the extract did not substantially alter the overall hydrophilic character of the starch–glycerol matrix. Similar results have been reported for films in which phenolic extracts were incorporated without markedly altering water retention, as the balance between hydrophilic and hydrophobic components may prevent significant changes in hygroscopicity.^42^

In contrast, DE-5% showed a significantly lower moisture content (*p* < 0.05), indicating reduced hygroscopicity after incorporation of the W/O/W emulsion. This reduction is attributed to the presence of the oil phase in the W/O/W emulsion, which likely reduces the availability of sorption sites for water and decreases the overall affinity of the film for moisture. A similar trend was reported by Zhao et al.^3^ for potato starch/PVA films containing clove-oil Pickering emulsion, where increasing emulsion concentration led to lower moisture content, with values ranging from 8.80 to 10.5%, reinforcing that structured hydrophobic phases can reduce water uptake in starch-based matrices.

WCA provides information about the wettability of films, as it results from the balance between the cohesive forces within water and the adhesive forces between water and the material surface. C film showed the lowest WCA value (*p* < 0.05), consistent with the highly hydrophilic surface due to the presence of free hydrophilic groups in starch (Table 4). The addition of free NE increased the WCA values, with no significant differences between NE-5% and NE-10% (*p* > 0.05), which is desirable for food-packaging applications, since higher WCA values indicate more hydrophobic surfaces.^2,45^ This may be attributed to the carotenoids present in the NE, which may have reduced surface polarity or become partially oriented toward the air interface during drying.^8^ In contrast, DE-5% showed a lower WCA than the NE-containing films. This indicates that the effect of the emulsion on surface wettability was not determined only by the lipid fraction itself, but also by changes in surface topography and heterogeneity, as suggested by the microscopy images. In this case, the presence of dispersed droplets may have altered the exposure of hydrophilic groups at the surface, leading to a more wettable material despite the presence of oil.^3^ A similar trend has been reported for biopolymeric films containing double emulsions, in which the incorporation of the emulsion reduced the water contact angle from 71.0 ± 3.6° in the control film to 6.0 ± 0.7° in the film containing double emulsion, indicating that these systems may increase surface wettability depending on matrix composition and surface reorganization during drying.^9^

A different behavior was observed for the solubility in water (Table 4). The C film showed the highest solubility (*p* < 0.05), confirming the highly hydrophilic nature of starch-based matrices.^22^ The incorporation of NE, at either 5% or 10%, resulted in a pronounced reduction in solubility, suggesting the formation of additional interactions between bioactive compounds and the starch chains, which may strengthen the biopolymeric network and limit water dissolution. Such decreases in solubility due to extract incorporation have been reported in gelatin-based films enriched with plant-derived polyphenols and in Persin gum-based films added with water-soluble carotenoids.^42,46^ The DE-5% exhibited solubility values similar to those of NE-containing films (*p* > 0.05), indicating that both NE addition and W/O/W emulsion incorporation improved resistance to dissolution. The hydrophobic contribution of the oil phase in the W/O/W emulsion likely plays a role in this reduction, acting as a physical barrier to water penetration, as previously observed by other authors.^5,9,46^

WVP is a critical property for packaging materials, since reducing moisture transfer is essential to maintain food quality and extend shelf life.^3^ In this study, C and NE-5% exhibited the highest WVP values (*p* < 0.05), whereas NE-10% and DE-5% showed lower permeabilities (Table 4). This result indicates that the lower extract level was insufficient to substantially modify the diffusion pathway through the matrix, while either increasing the extract concentration or incorporating it through a W/O/W emulsion led to a more resistant structure to water vapor migration.^47^ Although WVP values were normalized by film thickness, the significantly lower thickness observed for NE-5% and NE-10% may still reflect differences in matrix compactness and microstructural organization, which can contribute to variations in the vapor transport behavior.

For DE-5%, this reduction can be attributed to the presence of dispersed emulsion droplets, which likely increased the complexity of the diffusion pathway and reduced the effective area available for water vapor migration through the continuous starch phase. In addition, the lower moisture content of DE-5% suggests a less water-plasticized matrix, which may also have contributed to reduced molecular mobility and lower permeability. A similar trend has been reported for biopolymer films containing double emulsions, in which WVP decreased (0.42–0.25 g mm/m^2^ h kPa) after emulsion incorporation, an effect attributed to the presence of soybean oil, which reduced the hydrophilicity of the film matrix.^9^ Thus, the lower WVP observed for DE-5% is better interpreted as the result of combined effects of lipid incorporation, matrix reorganization, and decreased water affinity.

#### 3.2.3 Morphology, Crystallinity, Thermal, and Mechanical Properties

The incorporation of free NE and W/O/W emulsion affected the internal organization of the starch-based films, as evidenced by the combined morphological, structural, thermal, and mechanical analyses (Figures 4b–d and 5). Surface SEM images showed that all films were continuous and free of visible cracks, indicating that neither the extract nor the emulsified system impaired film formation. However, compared with the control film, the films containing NE and especially DE-5% exhibited rougher surfaces, suggesting that both the extract and the dispersed emulsion droplets altered matrix organization during drying. In the case of DE-5%, this effect may be associated with the presence of oil droplets and their partial migration during solvent evaporation, as previously reported for Persian gum-based films incorporating W/O/W emulsions encapsulating crocin and cinnamaldehyde.^46^ However, no phase separation was observed, indicating a good dispersion of the W/O/W emulsion within the starch biopolymeric matrix. In gelatin/chitosan-based films activated with W/O/W emulsions encapsulating pitanga leaf extract^9^ or *Pulicaria jaubertii* extract,^5^ the migration of oil to the film surface was more evident.

**Figure 5:**
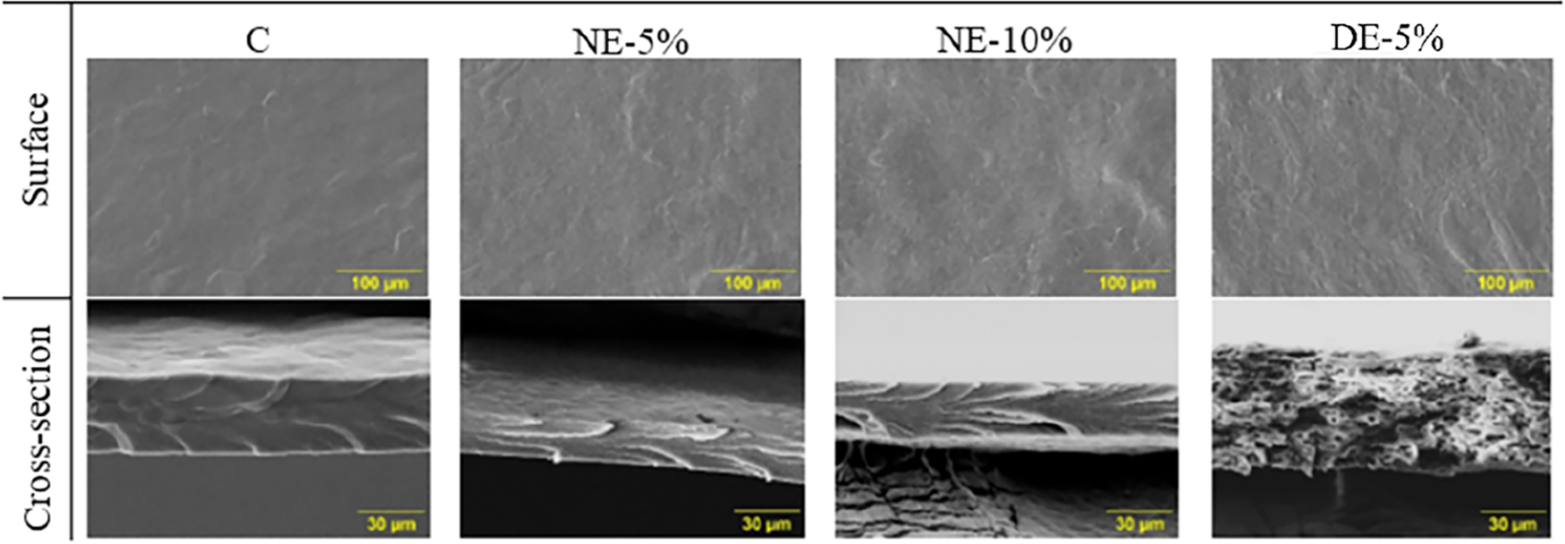
SEM images from surface and cross-section starch-based films without (C) and with NADES–based annatto seed extract at 5% (NE-5%) and 10% (NE-10%) or W/O/W emulsion at 5% (DE-5%).

Cross-sectional images provided additional evidence of these structural differences. While NE-5% and NE-10% remained relatively compact and did not show marked internal discontinuities, DE-5% exhibited pores and heterogeneities throughout the film thickness (Figure 5). Rather than indicating poor film formation, this result suggests that the incorporation of the W/O/W emulsion introduced dispersed domains into the continuous starch matrix, generating a less-compact internal structure. Similar microporous features have been reported for starch/PVA films containing Pickering emulsions, as well as for other biopolymer films containing double emulsions, in which dispersed lipid phases modified matrix packing without preventing film formation.^3,9^ These observations are relevant because they help explain the differences later observed in barrier and mechanical properties.

The diffractograms and relative crystallinity (RC) of all films were used to investigate their structural characteristics (Figure 4b). All films exhibited the typical semicrystalline character of starch-based materials, with amorphous and crystalline regions coexisting in the matrix. However, a noticeable decrease in the intensity of the crystalline peaks, particularly in NE-5%, NE-10%, and DE-5%, as well as the lower relative crystallinity values, indicates that the incorporation of these components hindered the formation of ordered amylose-rich domains. The peaks at 5.7°, 17.0°, 19.5°, and 21.7° (2θ), which are associated with the crystalline structure of amylose,^44^ are significantly affected by this reduction, suggesting that both the extract and the emulsified system interfered with chain rearrangement during drying. The Relative Crystallinity (RC) values further support this observation, with C exhibiting the highest crystallinity, followed by films with added components. This decrease in crystallinity suggests that the incorporation of NE and W/O/W emulsion interferes with the crystallization process of amylose,^2^ likely by disrupting chain mobility or causing steric hindrance, preventing the starch from forming well-ordered crystalline structures.^3,22^ Similarly, Zhao et al.^3^ observed that the addition of Pickering emulsion to polymer films resulted in a masking effect that eliminated the characteristic peak, indicating that the emulsion had an insignificant influence on the crystalline structure of the film.

The observed decrease in crystallinity can be attributed to the impact of NE and W/O/W emulsion on the internal structure of the starch matrix. These components, particularly the lipophilic emulsion, likely alter the interactions between the starch molecules, hindering the formation of ordered crystalline regions, as observed by Reis et al.,^20^ who reported similar X-ray diffraction (XRD) patterns with peaks at 2θ = 21.2° and 23.3° in propolis wax films. This change in crystallinity supports the idea that emulsions and their interactions can disrupt crystalline structures.

The thermal behavior of the starch-based films was evaluated by DSC using first and second heating scans (Figure 4c), allowing for distinction between irreversible thermal events related to processing history and intrinsic thermal transitions of the polymeric system. The first heating scan reflects the thermal history of the films, including melting of residual crystalline regions and relaxation phenomena induced during drying, whereas the second heating scan provides information on thermal transitions after elimination of prior thermal history, particularly glass transition events.^22,48^

For all formulations, two glass transition temperatures were identified. The first glass transition (Tg_1_), observed at low temperatures, is attributed to the plasticizer-rich phase (glycerol/water domains), while the second glass transition (Tg_2_), detected around 60–63 °C, corresponds to the starch-rich amorphous phase. This dual-Tg behavior is typical of plasticized starch-based films and arises from phase heterogeneity associated with partial miscibility between starch and low-molecular-weight plasticizers.^22,48^ The incorporation of NE or W/O/W emulsion did not significantly affect Tg_2_ values (Table 5), indicating that the molecular mobility of the starch backbone was not markedly altered.

**Table 5:** Thermal Properties (Tg_1_ and Tg_2_): Glass Transition Temperatures of Glycerol and Starch, Respectively; Tm_1_, Tm_2_ and Tm_3_: Melting Temperatures of Oil, Starch and NADES, Respectively; ΔHm_1_, ΔHm_2_ and ΔHm_3_: Melting Enthalpy of Oil, and Mechanical Properties of Starch-based Films without (C) and with NADES–based Annatto Seed Extract at 5% (NE-5%) and 10% (NE-10%) or W/O/W Emulsion at 5% (DE-5%)^a^,^b^

thermal properties	C	NE-5%	NE-10%	DE-5%
1st scan				
Tg_1_ (°C)	–75.22	-	-	-
Tg_2_ (°C)	61.65	61.09	61.57	61.79
Tm_1_ (°C)	-	-	-	–27.56
ΔHm_1_ (J/g)	-	-	-	2.19
Tm_2_ (°C)	82.59	83.73	85.27	83.6
ΔHm_2_ (J/g)	5.99	4.83	1.49	1.53
Tm_3_ (°C)	-	124.31	120.94	-
ΔHm_3_ (J/g)	-	9.99	7.03	-
2nd scan				
Tg_1_ (°C)	–80.58	-	-	-
Tg_2_ (°C)	63.29	61.33	61.56	61.61
mechanical properties	C	NE-5%	NE-10%	DE-5%
tensile strength (MPa)	20.85 ± 0.85b	20.70 ± 0.96b	31.32 ± 0.02a	21.84 ± 0.97b
elongation at break (%)	4.02 ± 0.07a	1.94 ± 0.10c	2.50 ± 0.07b	1.67 ± 0.02d
elastic modulus (MPa)	6.59 ± 0.24c	10.12 ± 0.94a	8.37 ± 0.01b	6.50 ± 0.21c

a Different letters on the same line indicate significant differences between C, NE-5%, NE-10% and DE-5% according to Tukey’s test (*p* < 0.05).

b Means ± standard deviation (*n* = 3).

An endothermic peak observed between 82 and 85 °C during the first heating scan is associated with the melting of residual crystalline regions of starch formed during film drying. This transition was no longer detected in the second heating scan, confirming that starch crystallites were irreversibly disrupted during the first thermal cycle and did not recrystallize upon cooling. This behavior is characteristic of gelatinized starch systems and has been widely reported for starch-based films produced by the casting method.^22,48^

Films containing free NE (NE-5% and NE-10%) exhibited an additional endothermic transition between 120 and 125 °C, which can be attributed to the melting of the CC/LA system (NADES). Previous studies have demonstrated that choline chloride-based NADES exhibit endothermic transitions associated with disruption of the hydrogen-bond network between hydrogen-bond donors and acceptors.^12^ In contrast, this transition was not observed for the DE-5% film, suggesting that encapsulation of the NADES within the W/O/W emulsion altered its thermal response, likely due to confinement within emulsion droplets.

The melting enthalpy associated with starch crystalline regions (ΔHm_2_) decreased significantly in films containing free extract and W/O/W emulsion compared with the C film, corroborating X-ray diffraction results that indicated a reduction in relative crystallinity. This decrease suggests that both the NE and the emulsified system interfere with starch chain organization, hindering the formation of ordered crystalline domains. Similar reductions in melting enthalpy and crystallinity have been reported for starch-based films incorporating plant extracts or emulsified lipid systems, due to disruption of starch–starch interactions.^3,48^

Overall, DSC results confirm that incorporation of NADES-based extracts and W/O/W emulsions modifies the thermal organization of the starch matrix without compromising its thermal stability within the temperature range relevant to food-packaging applications.

The enthalpy of the starch melting transition (ΔHm_2_) helps to clarify the molecular arrangement of the film matrix. A decrease in this value is observed for the films containing the NE and the W/O/W emulsion when compared with the C film. This may be explained by the interference of the plasticizers in the starch network, which reduces the crystalline fraction of the films.^2^ This hypothesis is supported by the RC obtained for the films. Similar trends have been widely reported for starch-based films incorporated with plant extracts or emulsified/encapsulated bioactive compounds, where the presence of low-molecular-weight compounds or oil droplets hinders starch chain rearrangement, leading to lower crystallinity and reduced melting enthalpy.^40,41,49^

Biopolymer films often show insufficient mechanical strength for real food packaging conditions, where materials must endure transport, storage, and handling, so additives such as plant extracts or dispersed lipid phases are explored to reinforce the polymer network and improve tensile performance.^4,42,50^ All formulations showed tensile strength values above the minimum typically required for flexible packaging applications of 11.7 MPa,^51^ indicating that the films retained mechanical integrity after incorporation of the active systems. Among the active films, NE-10% showed the highest tensile strength (Table 5), suggesting that the higher concentration of free extract promoted a more cohesive matrix, possibly due to additional intermolecular interactions and a higher total solids content.^4^ A similar strengthening effect has been reported for canna starch-based edible films, in which the addition of plant extracts directly increased mechanical strength, as higher extract contents raised the total solids in the film matrix, promoting a more compact and homogeneous polymer network and resulting in increased tensile strength.^52^

In contrast, DE-5% did not show the same reinforcing effect despite its improved barrier-related properties. This result is consistent with the microstructural observations, since dispersed lipid domains and internal heterogeneities can interrupt stress transfer through the continuous polysaccharide phase and therefore limit tensile reinforcement.^6^

Elongation at break was reduced in all active films relative to that of the C film, with DE-5% showing the lowest extensibility. This suggests that both NE and W/O/W emulsion incorporation reduced chain mobility or introduced structural heterogeneities that made deformation more difficult.^6,9^ For DE-5%, this effect was likely intensified by the lower moisture content since water acts as a plasticizer in starch matrices.^22,53^ Thus, the lower extensibility of DE-5% is consistent with a matrix that is simultaneously less hydrated and more structurally heterogeneous.^22,45^ This interpretation is supported by the SEM results and the lower moisture content previously discussed.

The elastic modulus values further support this interpretation. NE-5% and NE-10% showed higher modulus values than C and DE-5%, indicating increased stiffness after the addition of NE. By contrast, DE-5% remained closer to the control, suggesting that the emulsion did not act as a reinforcing filler. Instead, the lipid droplets appear to have behaved as dispersed soft domains within the matrix, which is consistent with previous reports for polysaccharide films containing emulsified lipid phases.^9^ Overall, these differences suggest that film formation and internal structure were also affected by the incorporation of the active phases. In this context, the reduced thickness observed for NE-5% and NE-10% may also indicate differences in film formation and matrix compaction during drying, which can indirectly influence the stress distribution and mechanical response.

Representative stress–strain curves are presented in the Supporting Information (Figure S2). All starch-based films exhibited relatively low elongation at break, with DE-5% and NE-5% showing the lowest values, suggesting reduced extensibility compared to that of the control and NE-10%. The control film exhibited the highest elongation among the formulations, indicating comparatively greater flexibility, which is also observable in the stress–strain curves. This behavior can be attributed to several factors. DE-5% displayed lower moisture content (Table 4), likely reducing chain mobility, while incorporation of NE and W/O/W emulsion also affected matrix organization, as suggested by the decreased relative crystallinity observed in XRD analysis (Figure 4b and Table 5).^3,22^ In addition, microstructural heterogeneities observed in SEM images may have contributed to earlier fracture under tensile stress.^6,52^ Overall, the combination of reduced hydration, lower crystallinity, and structural heterogeneity is consistent with the lower elongation observed for DE-5% and NE-5%.

Overall, the combined SEM, XRD, DSC, and mechanical results indicate that incorporation of free NADES–annatto extract and W/O/W emulsion modifies the internal organization of the starch matrix, affecting crystallinity and mechanical response without compromising structural integrity.

#### 3.2.4 Functional Groups by Fourier-Transform Infrared Spectroscopy (FTIR)

The FTIR spectra of C film, NE-5%, NE-10%, and DE-5% (Figure 4d) provide additional evidence of changes in the chemical environment of the starch-based matrix after incorporation of the extract and the W/O/W emulsion. The broad absorption band between 3600 and 3000 cm^–1^ is attributed to the stretching vibration of O–H groups, which are commonly associated with hydroxyl groups from starch, glycerol, and absorbed water.^3,22,45^ The lower intensity of this band in films containing NE and especially DE-5% may indicate changes in hydrogen-bond interactions within the matrix, which is consistent with the differences observed in moisture content and structural organization, as similarly observed by Devi et al.^50^ and Liu et al.^48^

At 2929 cm^–1^, the band assigned to C–H stretching vibrations decreased in intensity after incorporation of the extract and showed a slight shift in DE-5%, suggesting changes in the local chemical environment caused by the presence of the emulsified phase.^20^ Additionally, this peak, commonly observed in starches, suggests similar hydrophobic interactions between starch and its additives. These results align with the findings of Maniglia et al.^22^ who reported such behavior in starch-based materials, and Liu et al.,^48^ who noted similar shifts in polymer blends containing hydrophobic groups.

The peak at 1747 cm^–1^, which was more evident in DE-5%, is attributed to the stretching of the C=O bond, typical of fatty acid esters present in the emulsion, confirming the presence of the dispersed oil component in the film. Similar peaks have been reported for films incorporating double emulsions, such as those containing soybean oil, highlighting the influence of emulsions on the carbonyl group stretching.^9,20^

The band around 1650 cm^–1^ is mainly associated with the bending vibration of absorbed or bound water (H–O–H) in the starch-based matrix. Its lower intensity in the active films suggests reduced water association or modified hydrogen-bond interactions, which is consistent with the lower moisture content observed for DE-5%, similar to the behavior seen in films containing water-binding components such as curcumin.^48^

The peak at 1420 cm^–1^, related to C–O and CH_2_- associated vibrations, decreased after incorporation of NE and W/O/W emulsion, indicating that these additions affected the chemical environment of the polymer network.^5^ At approximately 1000 cm^–1^, corresponding to the C–O–C stretching vibration of starch glucose units, the C film showed the highest intensity, while the active films exhibited changes in band profile and intensity. Upon the addition of the NE, this peak weakened, and the W/O/W emulsion led to a slight increase in intensity, suggesting changes in the local organization of the starch network and in its interactions with the emulsified lipid phase.^48^ Similar trends have been observed in polysaccharide-based films, where the incorporation of emulsions alters the intensity and shape of the 1000 cm^–1^ peak.^3^

#### 3.2.5 Antioxidant Activity

Antioxidant activity is crucial for active biodegradable packaging since natural extracts incorporated into polymer matrices can scavenge reactive species, delay oxidation, and help preserve food quality during storage.^4,42^ When comparing the antioxidant activity of films with the activity of the NE alone, a clear reduction was observed for the NE-5% and NE-10% in both ABTS and DPPH assays (Figure 6).^8^ This result indicates that direct incorporation of the extract into the starch matrix did not fully preserve its radical scavenging capacity. Such a decrease is expected, since free phenolic and carotenoid compounds may undergo partial degradation during film formation and interact with polymer networks, which can reduce their mobility and limit their effective radical-scavenging capacity within the starch matrix, as widely reported in the literature.^4^

**Figure 6:**
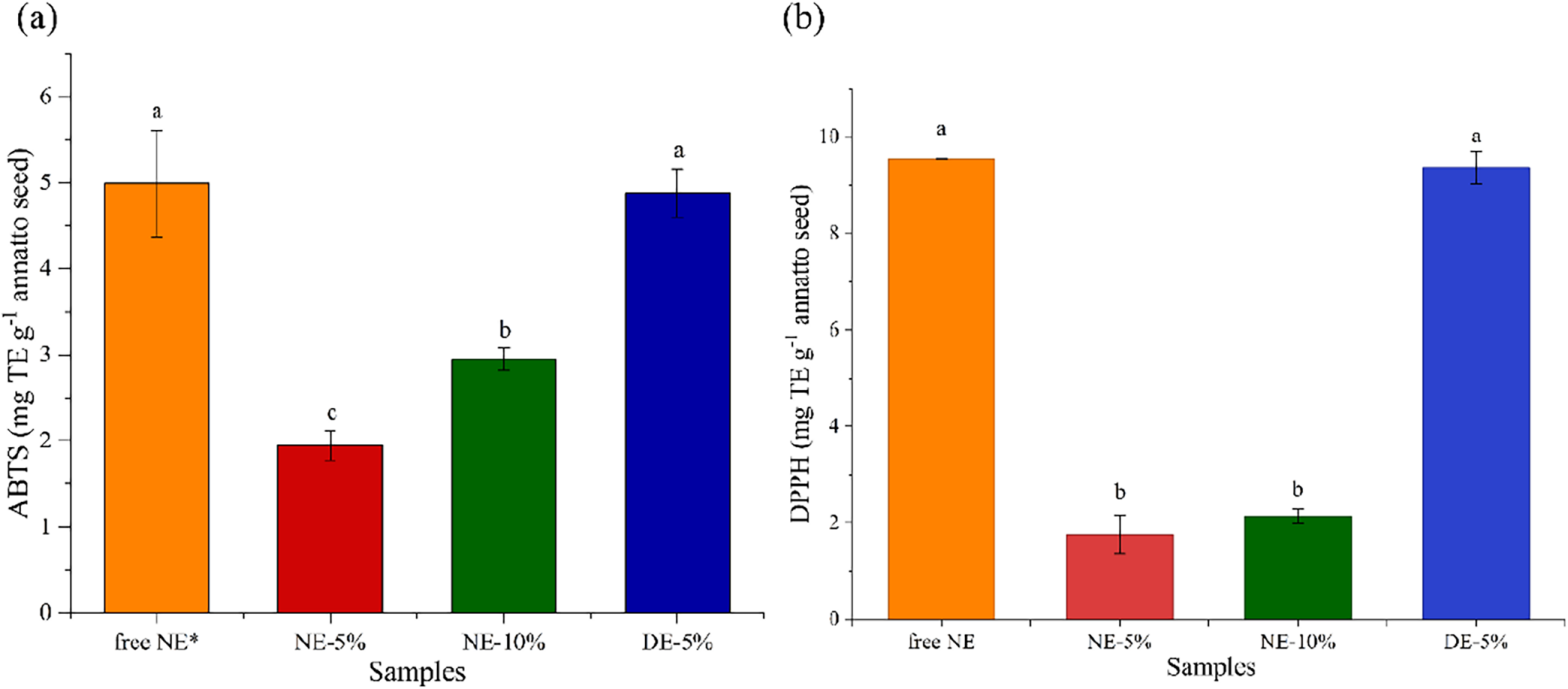
(a) ABTS antioxidant activity and (b) DPPH antioxidant activity of NADES–based annatto seed extract (NE) and starch-based films with NE at 5% (NE-5%) and 10% (NE-10%) or W/O/W emulsion at 5% (DE-5%). *ABTS antioxidant activity of free NE as reported by Balabram et al.^8^ Different letters indicate significant differences between NE, NE-5%, NE-10% and DE-5% according to Tukey’s test (*p* **<** 0.05).

While the ABTS assay revealed a significant increase in antioxidant activity when the extract concentration was increased from 5% to 10%, no significant differences between NE-5% and NE-10% were observed in the DPPH assay. This discrepancy may be attributed to the different mechanisms and sensitivities of the two methods, since ABTS is generally suitable for evaluating both hydrophilic and lipophilic antioxidants, whereas DPPH is more dependent on the accessibility and diffusion of antioxidant compounds within the polymer matrix.^6,21^

In contrast, DE-5% exhibited markedly higher antioxidant activity than the films containing free NE and showed values statistically similar to those of the free extract in both the ABTS and DPPH assays. This result suggests that the incorporation of the extract through the W/O/W emulsion improved the preservation of antioxidant functionality during film formation. A plausible explanation is that the emulsion reduced the direct exposure of sensitive compounds to the starch matrix and to processing conditions, thereby limiting degradation and unfavorable interactions. Similar behavior has been reported for active films containing encapsulated bioactive compounds, in which emulsified or Pickering systems retained higher antioxidant activity than the corresponding nonencapsulated forms.^6^ Comparable trends have also been observed for systems containing curcumin and pitanga leaf extract, where encapsulation helped preserve radical-scavenging capacity after incorporation into biopolymer matrices.^6,9^

From an application perspective, the combination of lower water solubility, lower moisture content, improved UV barrier, and higher antioxidant activity suggests that the DE-5% formulation may be particularly useful for developing active packaging for foods susceptible to light- and oxygen-induced deterioration, such as nuts, lipid-rich snacks, dehydrated products, and other foods.

## 4 Conclusion

This study showed that the incorporation of a NADES-based annatto seed extract through a W/O/W emulsion was an effective strategy to preserve antioxidant functionality and to improve the performance of potato starch-based films. Compared with the direct addition of the extract, the emulsified system resulted in films with lower moisture content and water vapor permeability, improved UV–Vis barrier properties, and higher antioxidant activity.

Morphological, diffraction, and thermal analyses indicated that the W/O/W emulsion modified the internal organization of the starch matrix, contributing to changes in the film microstructure and crystallinity without impairing film formation. Although the emulsion did not improve mechanical reinforcement, it contributed to barrier-related and functional properties that are relevant to active biodegradable packaging. The results also suggest that combining NADES extraction with W/O/W encapsulation is a useful approach for incorporating sensitive bioactive extracts into hydrophilic starch-based matrices. Collectively, these findings indicate that W/O/W emulsions are a promising strategy for incorporating NADES-derived bioactive compounds into hydrophilic starch-based matrices and for improving the functional performance of these materials. This approach may be particularly useful for the development of active packaging intended for foods susceptible to light- and oxidation-related deterioration.

Although the results demonstrate promising functional and structural properties, further studies evaluating the performance in real food systems and under storage conditions are required to confirm the practical applicability of these materials.

## Supplementary Material



## Notes

Residual plots of apparent viscosity versus shear rate for W/O and W/O/W emulsions (Figure S1); stress–strain curves of starch-based films (Figure S2) (PDF)
