# Supplementary material for: W/O/W Emulsions as a Strategy to Preserve NADES-Derived Annatto Carotenoids and Enhance the Functional Properties of Potato Starch-Based Active Packaging Films
Source: ACS Omega. 2026 Jul 31;11(31):46827–44. doi: 10.1021/acsomega.6c04826 (PMC13470849; doi:10.1021/acsomega.6c04826)
Supplement: Supplementary file 1 [file ao6c04826_si_001.pdf]

W/O/W emulsions as a strategy to preserve NADES-derived annatto carotenoids and enhance the functional properties of potato starch-based active packaging films

Sara K. Balabram [a], Fernanda P. Silva [a], Bianca C. Maniglia [a], Larissa Tessaro [a*]

[a]*University of São Paulo, São Carlos Institute of Chemistry, Av Trabalhador São Carlense, 400, 13566-590 São Carlos, SP, Brazil.*

*Corresponding author: larissa.tessaro@usp.br

Supporting Information for Publication

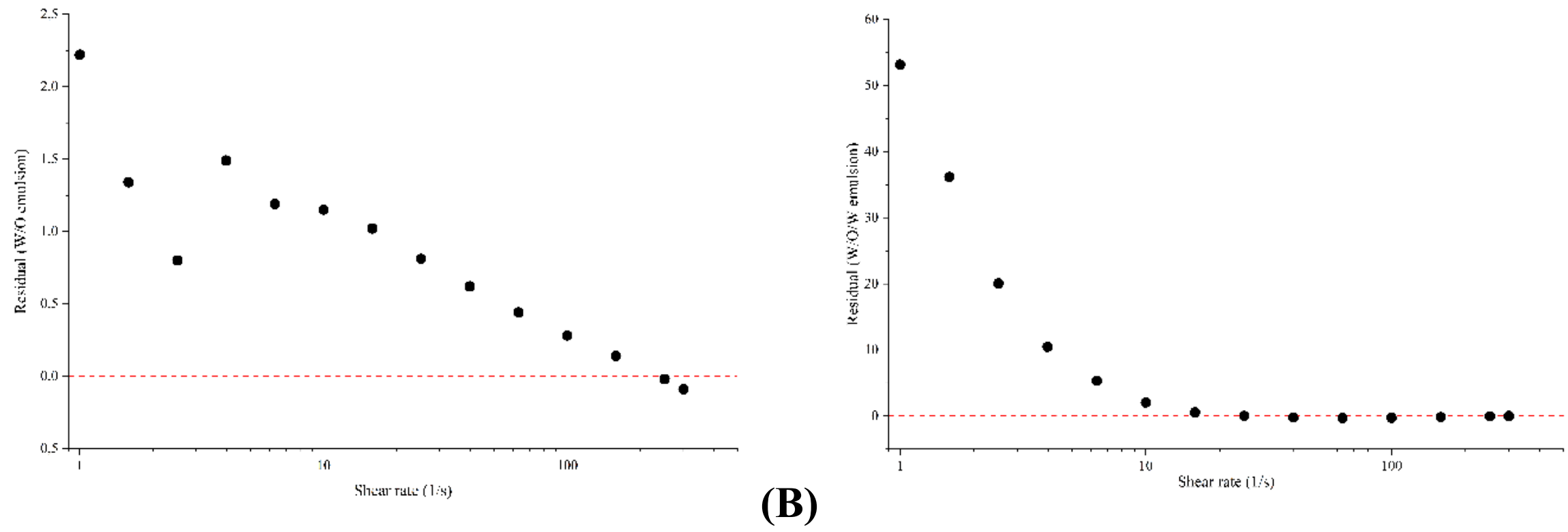

(A) **(B)**

Figure S1. Residual plots of apparent viscosity versus shear rate for emulsions: (A) W/O emulsion (Newtonian), (B) W/O/W emulsion (Power-Law).

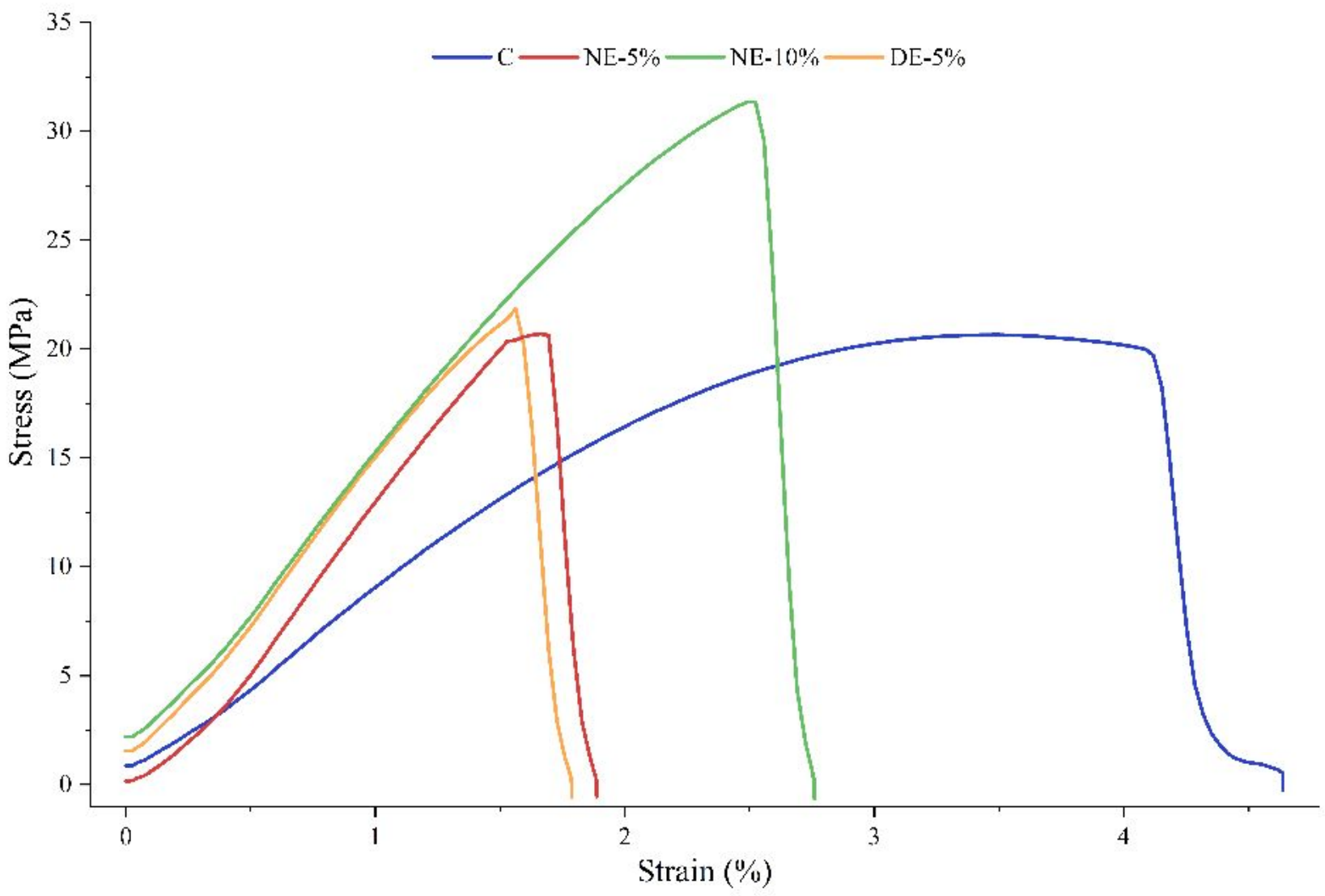

Figure S2. Stress–strain curves of starch-based films without (C) and with NADES-based annatto seed extract at 5% (NE-5%) and 10% (NE-10%) or W/O/W emulsion at 5% (DE-5%).
